# Self-Assembly 3D Porous Crumpled MXene Spheres as Efficient Gas and Pressure Sensing Material for Transient All-MXene Sensors

**DOI:** 10.1007/s40820-022-00796-7

**Published:** 2022-02-05

**Authors:** Zijie Yang, Siyuan Lv, Yueying Zhang, Jing Wang, Li Jiang, Xiaoteng Jia, Chenguang Wang, Xu Yan, Peng Sun, Yu Duan, Fangmeng Liu, Geyu Lu

**Affiliations:** 1 State Key Laboratory of Integrated Optoelectronics, College of Electronic Science and Engineering, Jilin University, 2699 Qianjin Street, Changchun, 130012 People’s Republic of China; 2 School of Electronic and Information Engineering, Changchun University of Science and Technology, Changchun, 130022 People’s Republic of China

**Keywords:** All-MXene, 3D porous crumpled MXene sphere, Transient, NO_2_ and pressure sensor

## Abstract

Environmentally friendly degradable sensors with both hazardous gases and pressure efficient sensing capabilities are highly desired for various promising applications, including environmental pollution monitoring/prevention, wisdom medical, wearable smart devices, and artificial intelligence. However, the transient gas and pressure sensors based on only identical sensing material that concurrently meets the above detection needs have not been reported. Here, we present transient all-MXene NO_2_ and pressure sensors employing three-dimensional porous crumpled MXene spheres prepared by ultrasonic spray pyrolysis technology as the sensing layer, accompanied with water-soluble polyvinyl alcohol substrates embedded with patterned MXene electrodes. The gas sensor achieves a ppb-level of highly selective NO_2_ sensing, with a response of up to 12.11% at 5 ppm NO_2_ and a detection range of 50 ppb–5 ppm, while the pressure sensor has an extremely wide linear pressure detection range of 0.14–22.22 kPa and fast response time of 34 ms. In parallel, all-MXene NO_2_ and pressure sensors can be rapidly degraded in medical H_2_O_2_ within 6 h. This work provides a new avenue toward environmental monitoring, human physiological signal monitoring, and recyclable transient electronics.

**Supplementary Information:**

The online version contains supplementary material available at 10.1007/s40820-022-00796-7.

## Highlights

- 3D porous crumpled MXene spheres were synthesized by ultrasonic spray pyrolysis technology.
- All-MXene transient sensors utilizing porous crumpled MXene sphere as sensing material and MXene films as electrodes were developed, which achieved excellent gas/pressure sensing performance.
- Both gas and pressure sensors can achieve rapid and controllable degradation in medical-grade H_2_O_2_ (2%) within 6 h.

**Supplementary Information:**

The online version contains supplementary material available at 10.1007/s40820-022-00796-7.

## Graphical abstract

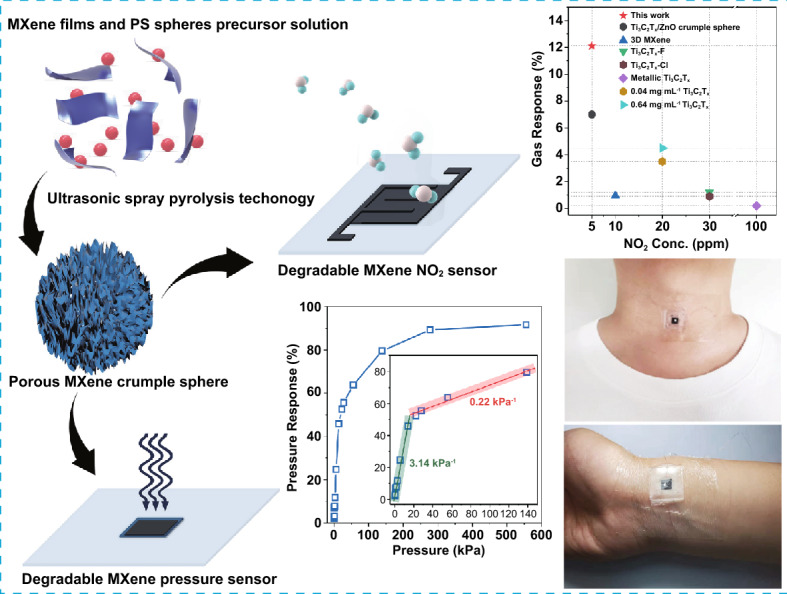

## Introduction

In the Internet of Things era, wearable sensors as information receiving nodes have grown rapidly [1]. Wearable gas and pressure sensors, in particular, are useful for detecting hazardous gases and monitoring human physiological signals [2, 3]. Meanwhile, transient and environmentally friendly devices that reduce the growing electronic waste are becoming crucial [4–6]. Therefore, the development of high-performance transient gas and pressure sensors has essential significance in wearable electronics.

Recently, transient gas and pressure sensors have been emerging [7, 8]. Jiang et al. developed a transient paper-based composite decorated with reduced graphene oxide and polyaniline with ammonia sensitivity and partial degradation [9]. Shen’s group demonstrated a bacterial cellulose/MXene composite aerogel toward ammonia and pressure detection [10]. However, the above-mentioned sensors only achieved partial degradation when realizing gas/pressure sensing. In this regard, traditional composite-based sensor materials struggle to meet the demands of high gas and pressure sensitivity and complete device transiency. Gas and pressure sensors made of the same degradable sensing and electrode materials are ideal for achieving full transiency upon only one external trigger. MXenes (Ti_3_C_2_T_*x*_), as a novel class of two-dimensional nanomaterials with rich surface functional groups, have been identified as the sensing layer and electrode due to their high conductivity, excellent signal-to-noise ratio, and abundant hydroxyl on the surface, which is superior to other metal oxides and two-dimensional (2D) materials [11–18]. Meanwhile, because of their chemical instability, MXenes exhibit controllable transiency in H_2_O_2_ and NaOH aqueous solutions [19, 20]. To achieve superior gas and pressure-sensing properties while maintaining complete degradation, it is necessary to tailor the architecture and composition of MXene sensing materials and ingeniously design the device architectures.

In MXene-based gas sensors, researchers have adopted different strategies to induce abundant oxygen terminals and high specific surface area, such as surface group modification [21, 22], microstructure design [23], and compounding with other nanomaterials [24], resulting in good sensitivity, selectivity, and low detection limits. In addition, the intercalation of various nanomaterials between MXene layers, such as silk fibroin [25, 26], cellulose nanofibers [19, 27], and holothurian-like microspheres [28], effectively enhances the piezoresistive properties. However, none of the above modification treatments are efficient toward the synergetic improvement of both the gas and pressure-sensing properties. Recent work has demonstrated that increasing the material porosity is a practical approach for boosting both gas and pressure sensing characteristics, attributed to the increased contact area between the sensitive layer and gas molecules as well as the compressibility of the sensing layer [29, 30]. 3D MXene spheres with the hollow porous structure meet the above requirements via multiple synergetic strategies. Compared with 2D MXene film, the anti-aggregation structure of porous 3D MXene spheres is very beneficial for gas sensing because of the minimized loss of specific surface area caused by the film aggregation [23, 31]. Porous MXene spheres have substantially more edge defects than MXene sphere, which has shown to significantly increase the gas adsorption capacity, particularly for NO_2_ [23]. Therefore, porous MXene spheres may have better NO_2_ selectivity than MXene spheres. Moreover, the hollow structure of the 3D MXene spheres could deform when exposed to external pressure, resulting in changes in electrical conductivity. As a result, all-MXene sensors based on a 3D MXene spheres with a porous hollow structure are expected to achieve high-performance gas and pressure sensing without composite other materials.

Herein, we developed transient all-MXene sensors with gas and pressure sensing capabilities, respectively. The transient gas and pressure sensors employ porous crumpled MXene spheres prepared by ultrasonic spray pyrolysis technology as the sensing layer and polyvinyl alcohol (PVA) substrate embedded with MXene slurry as the electrodes. This fully transient sensor outperforms state-of-the-art studies regarding ultra-wide pressure detection range and ultra-high sensitivity toward NO_2_. This work provides another way along the route to wearable and recyclable transient electronics.

## Experiment

### Preparation of Ti_3_C_2_T_***x***_ MXene Colloid

Ti_3_C_2_T_x_ MXene colloids were synthesized by etching the Ti_3_AlC_2_ phase (Jilin 11 Technology Co., Ltd., China) with LiF/HCl as reported previously [23]. First, 1 g LiF (Aladdin, > 99.99%) was added into 40 mL 12 M HCl (Xilong Scientific Co., Ltd.) and stirred in a water bath of 40 °C for 10 min. After LiF was completely dissolved, 1 g Ti_3_AlC_2_ was slowly added to the mixed solution, and then, a 40 °C water bath was conducted for 24 h. After the reaction, the product was centrifugally washed until the supernatant became neutral. Next, the clay Ti_3_C_2_T_*x*_ was dispersed in an ice bath by ultrasonic treatment for 1 h. Finally, a small amount of Ti_3_C_2_T_*x*_ dispersion was extracted to make the Ti_3_C_2_T_*x*_ membrane by vacuum filtration to determine the concentration.

### Preparation of Polyphenylene (PS) Sphere Colloid

PS spheres were synthesized by an emulsion-free polymerization method [32]. In brief, 30 mL styrene (Xilong Scientific Co., Ltd.) and 0.25 g poly(sodium 4-styrene sulfonic acid) (Aladdin, Mw: ~ 70,000), and 0.15 g sodium bicarbonate (Aladdin) were dissolved in 300 mL deionized water and stirred in an oil bath of 70 °C for 1 h. 0.15 g potassium persulfate (Aladdin, > 99.99%) was then added into the solution, and the solution was stirred in a 70 °C oil bath under N_2_ atmosphere for 6 h. After the reaction, the white precipitate was thoroughly washed by high-speed centrifugation. Finally, the white precipitate was uniformly dispersed in deionized water using a column ultrasonic machine to obtain a colloid of PS spheres. Similarly, a small amount of PS spheres colloid was extracted to make PS spheres membrane by vacuum filtration to determine the concentration.

### Preparation of Porous Crumpled MXene Sphere

Porous crumpled MXene spheres were synthesized by ultrasonic spray pyrolysis technology. Taking 2–5 as an example, the prepared Ti_3_C_2_T_x_ MXene colloid (10 mg mL^−1^) and 5 mL PS sphere colloid (48 mg mL^−1^) were mixed and diluted by adding deionized water to prepare 50 mL ultrasonic spray precursor solution, in which the concentration of Ti_3_C_2_T_x_ MXene was maintained at 2 mg mL^−1^. The prepared precursor solution was put into the atomization chamber. The fine water mist generated by ultrasound was brought into the tubular furnace preheated to 800 °C through Ar gas. Finally, the MS-2-5 powder generated was collected by the electrostatic collector at the back end. For MS-2-10 and MS-2-20, the only difference was that 10 and 20 mL of PS sphere colloid were added to the precursor solution, respectively.

### Characterizations

The morphology of porous crumpled MXene sphere observed by field-emission scanning electron microscopy (FESEM; JEOL JSM-7500F) with an acceleration voltage of 5 kV and transmission electron microscopy (TEM; JEM 2100 F) with an acceleration voltage of 200 kV. The X-ray diffraction (XRD) patterns of the porous crumpled MXene sphere powder were analyzed using Rigaku D/Max 2550 with Cu Kα radiation (*λ* = 1.5418 Å) in the 2*θ* range of 3°–80°. The surface characteristics of the porous crumpled MXene sphere powder were measured using an ESCALAB 250 X-ray photoelectron spectrometer (XPS) with an X-ray source (Al Kα *hυ* = 1486.6 eV). The specific surface area and pore size distribution of the prepared porous crumpled MXene sphere powder were determined from nitrogen adsorption/desorption isotherms by Brunauer–Emmett–Teller (BET, Micromeritics Gemini VII).

### Fabrication and Performance Measurement of the Transient NO_2_ Sensor

The prepared Ti_3_C_2_T_x_ MXene colloid was centrifuged at 20,000 rpm for 30 min, and the MXene slurry was precipitated. Masked by the hydrocoagulant film, the MXene slurry was scraped on the glass mold (square groove specification: 21 × 21 × 2 mm^3^) to form a cross-finger electrode pattern. The electrode width and electrode spacing were all 1 mm. After the MXene slurry was dried, PVA (0588 low-viscosity) aqueous solution with a concentration of 15 wt% was slowly dropped into the prepared solution. After natural drying, the PVA film with MXene cross-finger electrode was peeled off. The electrode was masked through the adhesive tape, and then, 5 mg porous crumpled MXene spheres powder was dispersed in 0.1 mL ethanol and finally dip-coated on the surface of the electrode. After natural drying and removing the mask, the NO_2_ sensor based on porous crumpled MXene spheres was successfully fabricated. The sensor was placed in the gas cavity of the self-made dynamic test system, where the different concentrations of the target gas were obtained by controlling the ratio of dry air (80% N_2_ and 20% O_2_) and the target gas, with the total flow rate of gas maintained at 500 sccm (standard cubic centimeter per minute). The real-time resistance change of the sensor was detected by connecting the digital multimeter (Fluke 8846A), and the built-in power supply of Fluke 8846A was 5 V for resistance measurement.

### Fabrication and Performance Measurement of the Transient Pressure Sensor

MXene top and bottom electrodes were made in the same way that MXene cross-finger electrodes were made. The square scratching pattern (6 × 6 mm^2^) and lead connection were different. After the MXene slurry was dried, the copper wires were connected to the MXene blocks using conductive silver slurry, and then, the PVA solution was poured. 20 mg porous crumpled MXene spheres powder was evenly dispersed in 2 mL MXene colloid (2 mg mL^−1^), and then, a round membrane with a diameter of 18 mm was obtained by vacuum filtration. The membrane was cut into a square (6 × 6 mm^2^) as the pressure sensing layer and placed between the top and bottom MXene electrodes. The square sensing layer was tightly encapsulated by brushing PVA aqueous solution on the top and bottom PVA membrane to produce a pressure sensor. Copper wire leads reserved in the top and bottom MXene square electrodes were used for output signals. The pressure-sensing properties were measured by the electromechanical universal testing machine (Mark-10), and the dynamometer model was M5-5. During the pressure test, the sensing area of the pressure sensor was fixed directly below the dynamometer probe about 1 cm^2^, and the probe completely covers the sensing area of a pressure sensor. The test conditions such as pressure, frequency, and press time can be adjusted by setting maximum force and maximum brake point. The real-time resistance change of the sensor was detected by connecting the digital multimeter (Keithley DMM6500). The pressure response (*R*_e_) was defined as |Δ*R*/*R*_0_|× 100%, where Δ*R* means the change of the stable resistance between pressure releasing and loading states, and R_0_ represented the stable resistance of sensor without pressure loading. In addition, *δR*_e_/*δP* was recorded as the sensitivity (*S*) of the pressure sensors, where P represented the intensity of the applied pressure.

## Results and Discussion

**Fig. 1:**
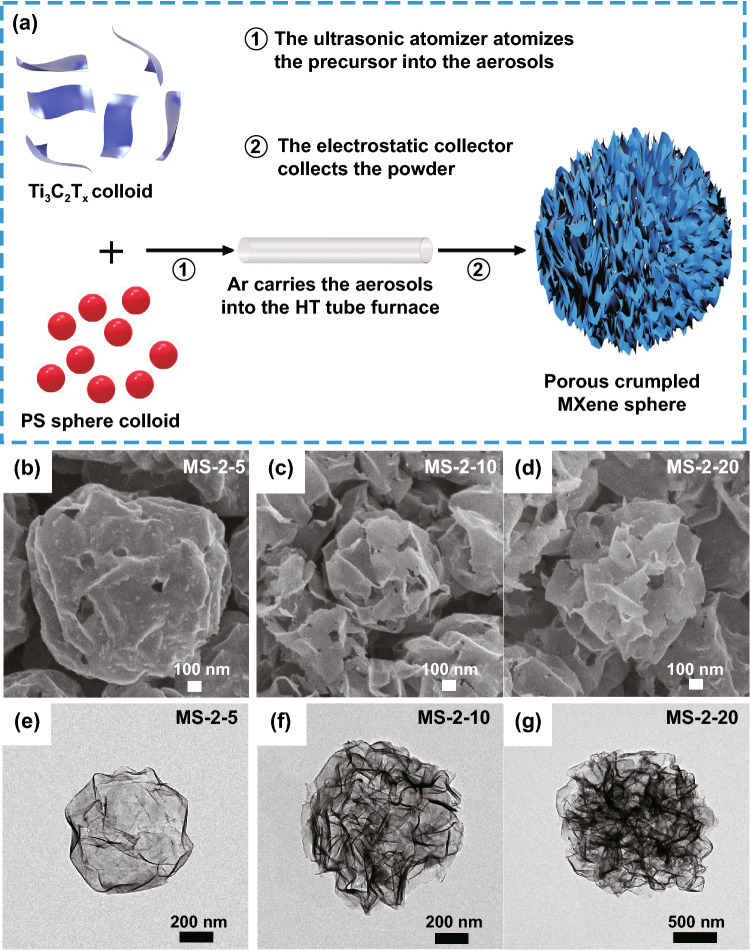
**a** The synthesis scheme of porous crumpled MXene spheres. SEM images of **b** MS-2-5, **c** MS-2-10, and **d** MS-2-20. TEM images of **e** MS-2-5, **f** MS-2-10, and **g** MS-2-20

The synthetic process of porous crumpled MXene sphere is displayed in Fig. 1a. MXene colloid and PS sphere colloid (grain diameter: ~ 250 nm, Fig. S1a–b) were evenly mixed in a particular proportion and then atomized into aerosols through an ultrasonic atomizer, which was immediately carried into a high-temperature tubular furnace through high-purity Ar gas. The aerosol encountered high temperatures in the tube furnace, and the moisture vaporized instantly. The disappearance of the internal stress caused the dispersed MXene films and PS spheres in the aerosol to collapse and accumulate inward. At the same time, PS spheres began to decompose when heated and produced CO_2_ gas, which broke through crumpled MXene spheres and formed a large number of holes on their surface. Porous crumpled MXene sphere powder generated in one step in the tube furnace was then collected by the back-end electrostatic collector. In order to achieve the best porous effect, different proportions of porous crumpled MXene spheres were synthesized by mixing PS spheres with different amounts of MXene, denoted as MS-2-5, MS-2-10, and MS-2-20, respectively.

SEM images of porous crumpled MXene spheres (Fig. 1b–g) indicated the surface morphology became wrinkled, and the size and number of surface holes increased with the increase of PS spheres. Specifically, the round and smooth state in MS-2-5 was transformed into many ridge-like humps on the surface in MS-2-10 and MS-2-20. The resulting ridge-like humps structure was formed by decomposing PS spheres in crumpled MXene spheres, leading to the internal collapse and rupture and holes formed on porous crumpled MXene spheres (Fig. S1f). As the proportion of PS spheres increased, these phenomena became more pronounced. However, when further increase of PS spheres to 20 mL, a large area of the porous crumpled MXene spheres would lose the supporting force, resulting in the collapse of the porous crumpled MXene spheres and the loss of 3D crumpled sphere structure (Fig. S1c–e).

**Fig. 2:**
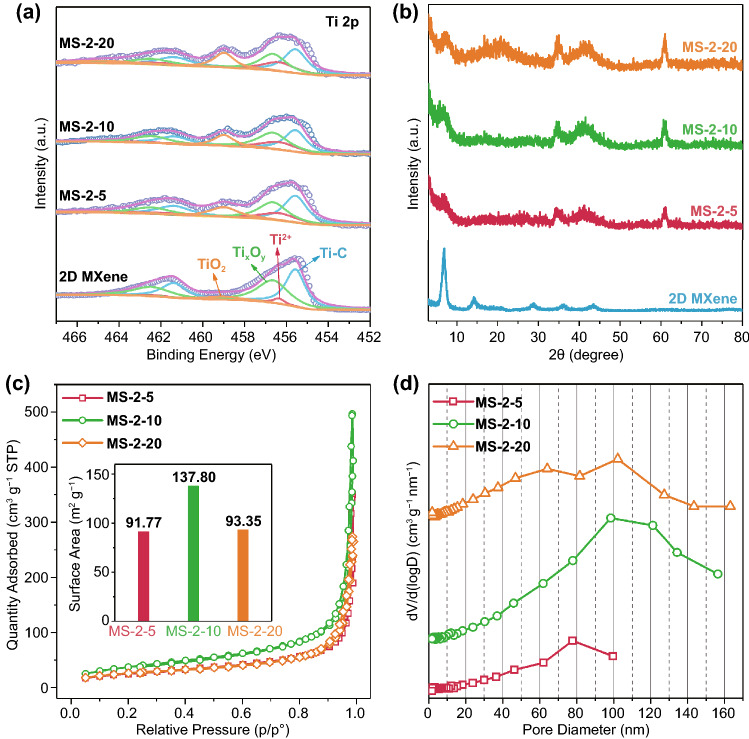
**a** Ti 2p XPS spectra and **b** XRD patterns of 2D MXene, MS-2-5, MS-2-10, and MS-2-20. **c** N_2_ adsorption–desorption isotherms and **d** pore size distribution of MS-2-5, MS-2-10, and MS-2-20

XPS was applied to reveal the chemical states and bonding configurations of porous crumpled MXene spheres. The XPS survey spectra of 2D MXene, MS-2-5, MS-2-10, and MS-2-20 displayed different Ti, C, O, and F elements signals in porous crumpled MXene spheres (Fig. S2a). The intensity of C 1 s peak and O 1 s peak rose with the proportion of PS spheres increased, demonstrating the decomposition of PS spheres and increase in oxygen terminals. In detail, we measured the high-resolution Ti 2p spectra of 2D MXene, MS-2-5, MS-2-10, and MS-2-20 in Fig. 2a. The Ti 2p spectra were spin splitting into Ti 2*p*_3/2_ and Ti 2*p*_1/2_ with a distance of 5.8 eV. Each splitting peak can be divided into four peaks at 455.57, 456.34, 456.64, and 458.98 eV, corresponding to the four states of Ti-C, Ti^2+^, Ti^3+^, and TiO_2_ [23]. The intensity of TiO_2_ peaks enhanced gradually with more PS spheres due to the increase in oxygen terminals and slight oxidation. The formation of porous crumpled MXene spheres was caused by the destruction of the complete lamellar structure, yielding more edges and defects on MXene. In high-resolution O 1 s spectra of 2D MXene, MS-2-5, MS-2-10, and MS-2-20 (Fig. S2b), each spectrum can be fitted from three peaks at 529.89, 531.86, and 533.23 eV, representing Ti–O, O–H, and C–O–C, respectively [33–35]. It can be seen that the proportion of –OH terminal in porous MXene crumpled spheres was significantly lower than that in 2D MXene film after high-temperature treatment.

XRD patterns of 2D MXene, MS-2-5, MS-2-10, and MS-2-20 are shown in Fig. 2b. The (002) characteristic diffraction peak of 2D MXene was strong, and the secondary diffraction peak was also prominent [23]. Combined with the XPS spectrum of 2D MXene, it could be indicated that the 2D MXene we prepared with high quality and purity. After forming a porous sphere structure, the intensity of (002) peak decreased significantly, and (110) peak became pronounced due to the increased stacking and random orientation of MXene films. The N_2_ adsorption/desorption isotherms of porous crumpled MXene sphere displayed typical mesoporous characteristics with the type III isotherm and a type H3 hysteresis loop (Fig. 2c). The isotherm had no inflection point, and the adsorption appeared self-accelerating phenomenon. Moreover, there was no apparent saturated adsorption platform under high pressure, indicating that the pore structure was irregular, consistent with the irregular holes observed in SEM. The pore size observed by SEM was also coincident with the pore size distribution analysis (Fig. 2d). The pore sizes of MS-2-20 and MS-2-10 were concentrated around 100 nm, while the pore sizes of MS-2-5 were mainly distributed around 80 nm. It should be noted that the specific surface area of MXene increases sharply with the generation of the porous sphere structure. The specific surface area of MS-2-5, MS-2-10, and MS-2-20 reached 91.77, 137.80, and 93.35 m^2^ g^−1^, respectively, compared with 33.56 m^2^ g^−1^ for the dried MXene films as previously reported [23]. The specific surface area of MS-2-20 did not further increase due to the collapse of the sphere structure with excessive PS spheres mentioned above in Fig. S1e.

**Fig. 3:**
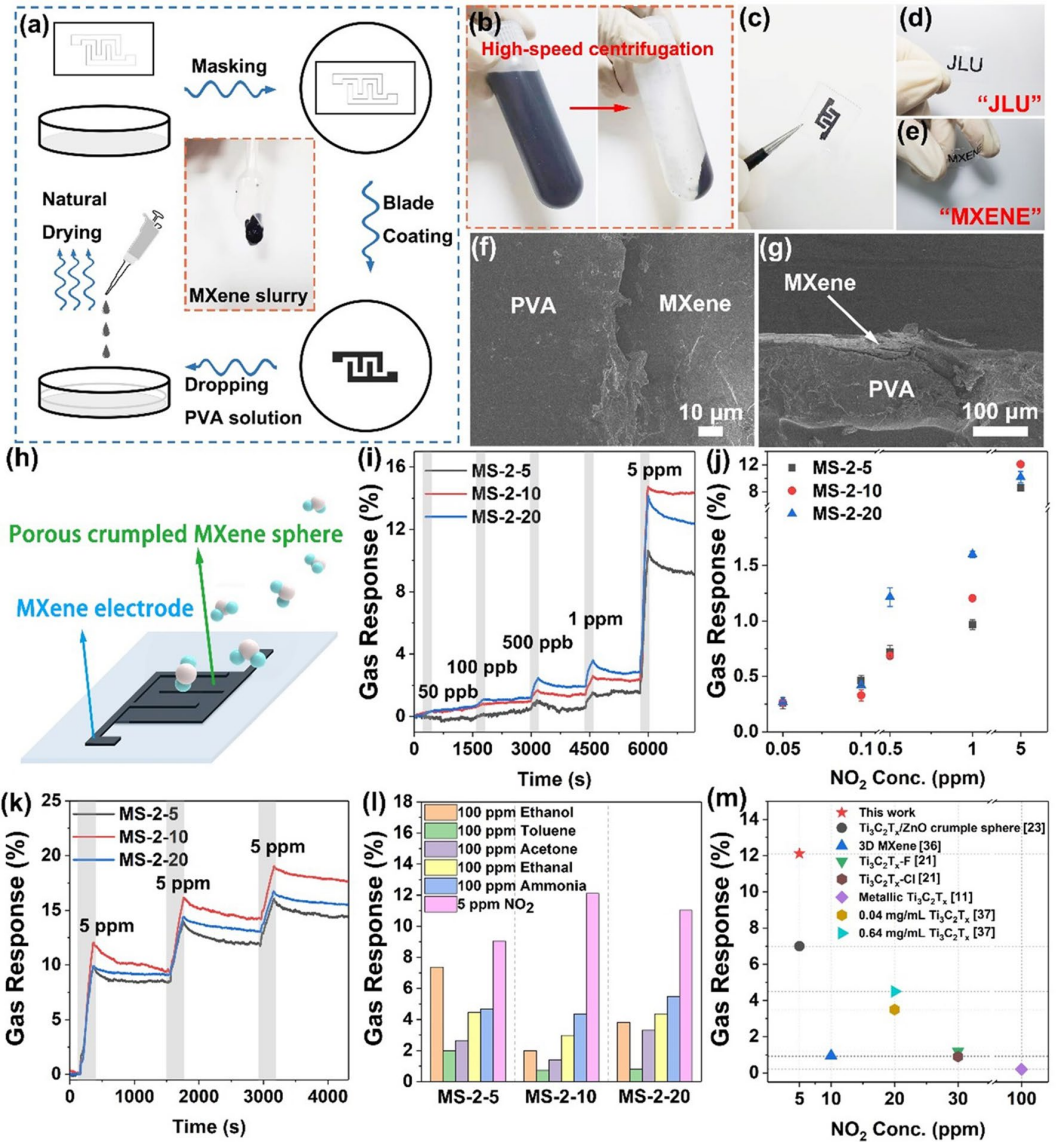
**a** Schematic diagram of the fabrication process of the transient PVA substrate with the MXene cross-finger electrode and the photograph of the MXene slurry. **b** Images of the preparation process of MXene slurry. **c** Image of the transient PVA substrate with MXene cross-finger electrode. **d**–**e** Images of the transient PVA substrate with conductive patterns of “JLU” and “MXENE.” **f** The frontal SEM image and **g** the sectional SEM image of the PVA substrate with MXene cross-finger electrode. **h** Schematic diagram of the transient NO_2_ sensor based on a porous crumpled MXene sphere. **i** Dynamic response–recovery curve of sensors based on MS-2-5, MS-2-10, and MS-2-20 in the different concentrations of dry NO_2_. **j** Gas response of sensors based on MS-2-5, MS-2-10, and MS-2-20 depending on NO_2_ concentration. **k** Real-time resistance curve of MS-2-5, MS-2-10, and MS-2-20 in response to 5 ppm of NO_2_ for three consecutive times. **l** The maximum resistance change rate of sensors based on MS-2-5, MS-2-10, and MS-2-20 to 100 ppm of ethanol, acetone, ethanol, toluene, NH_3,_ and 5 ppm of NO_2_. **m** Comparison of the performance of MXene-based NO_2_ sensors

The fabrication process of the transient substrate with the MXene interdigital electrode is shown in Fig. 3a. MXene slurry was readily produced by high-speed centrifugation (Fig. 3b), yielding a single layer mixture and few MXene films. The interdigital electrode pattern was fabricated by scraping and coating the MXene slurry on the bottom of the glass mold through the hydrogel mask. Subsequently, PVA aqueous solution (15 wt%) was coated onto the dried MXene patterned electrode. After dried at room temperature for 24 h, the PVA film with MXene cross-finger electrode (Fig. 3c) was peeled off and clipped to obtain the substrate. We also prepared PVA films with conductive patterns of "MXENE" and "JLU," as shown in Fig. 3d–e, and the detailed production steps are displayed in Fig. S3. As evidenced by the excellent conductivity (15.1 Ω) of the MXene electrode (Fig. S4a), we confirmed its feasibility as an electrode. The stability of MXene electrodes was tested with polyimide tape (Fig. S4b–d). After removing the adhesive tape, MXene electrodes could stably adhere to the substrate with a resistance change rate of only 1.3%. As shown in Fig. 3f–g, MXene electrodes were embedded in the PVA membrane, enabling robust and highly stable MXene electrodes on the PVA substrate.

The transient NO_2_ sensors were fabricated by drop-casting porous crumpled MXene spheres powder on the prepared water-soluble PVA substrates embedded with interdigital MXene electrodes (Fig. 3h). The gas-sensing properties were tested by a dynamic test system (Fig. S5). We measured the performance of porous crumpled MXene spheres with different ratios in continuous response to 50 ppb, 100 ppb, 500 ppb, 1 ppm, and 5 ppm NO_2_ in Fig. 3i. The transient sensor based on MS-2-5 had higher noise and the lowest responses at each concentration among three porous crumpled MXene spheres, while the sensor based on MS-2-20 performed best at a low concentration of NO_2_. The above results were confirmed by the response values corresponding to different concentrations of NO_2_ shown in Fig. 3j. When the concentration of NO_2_ reached 5 ppm, the response value of the MS-2-10 sensor remained the highest among the three kinds of porous spheres in multiple tests, reaching 12.11% at most, which was a considerable improvement compared with previous gas sensors based on MXene film [11, 23]. Meanwhile, the sensor based on MS-2-10 had a low detection limit of 50 ppb with a response of 0.25%.

The repeatability of the sensors was measured by exposing the sensors to 5 ppm NO_2_ three consecutive times (Fig. 3k). It could be seen that the MS-2-10 sensor maintained the highest response with almost the same response values three times in Fig. 3c. It had an irreversible response to each concentration of NO_2_ and could maintain the initial response when out of the gas atmosphere to be measured, making sensors very suitable as disposable and discardable NO_2_ sensors. In Fig. S6, the selectivity of the sensors was investigated by comparing the response of the sensors to 100 ppm ammonia and various volatile organic compounds (VOCs). Although the concentration of the contrast gases was much higher than 5 ppm, the response of the sensors based on porous crumpled MXene spheres was much higher to 5 ppm NO_2_ than to ammonia and various VOCs. Obtained from Fig. 3l, the MS-2-10 sensor showed the best NO_2_ selectivity among the sensors based on porous crumpled MXene spheres, and its responses to ammonia, ethanol, acetone, ethanol, and toluene were 4.35%, 1.99%, 1.39%, 2.98%, and 0.73%, respectively. It was noteworthy that the MS-2-10 sensor exhibited much higher gas response and lower detection limits than those of the sensors based on MXene films reported in previous work (Fig. 3m) [11, 21, 23, 36, 37]. The effect of relative humidity on NO_2_ sensing performance was also investigated at the relative humidity of 0%, 30%, 60%, and 90%, respectively (Fig. S7). The response of MS-2-10 to NO_2_ was improved as humidity increased, indicating that water molecules can promote the adsorption of NO_2_ molecules to the MXene surface. The excellent gas-sensing performance of MS-2-10 was attributed to its large specific surface area, as well as an abundance of edges and defects generated by folding and porous structure, as previously discussed [23]. MS-2-10 has many adsorption sites for gas molecules because of its large specific surface area, while the rich edge defects greatly enhanced the adsorption capacity of gas molecules, especially for NO_2_ molecules. The electron transfer between the surface of MXene and NO_2_ molecules led to the decrease in carrier concentration and electrical conductivity.

**Fig. 4:**
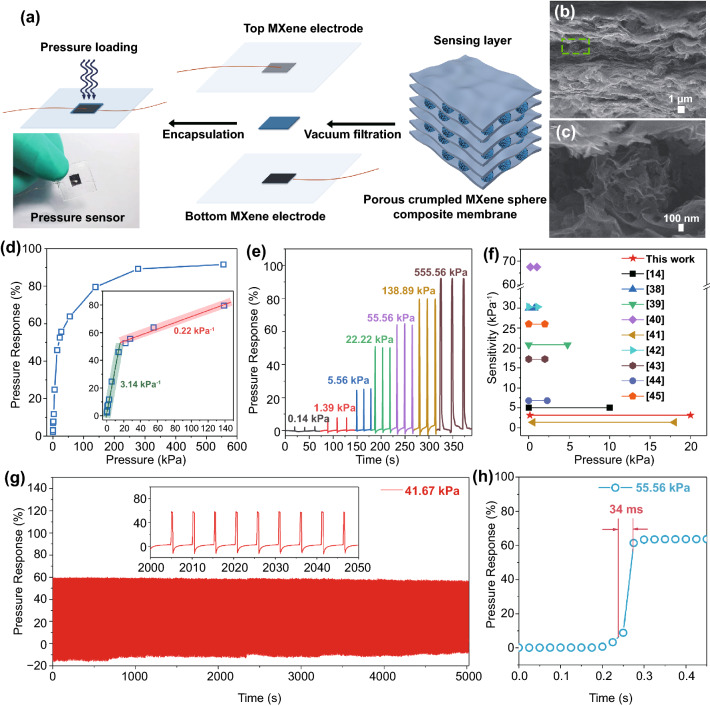
**a** Schematic diagram of the pressure sensor fabrication based on MS-2-10 and a photograph of the fabricated pressure sensor. **b** The cross section SEM image of the composite membrane and **c** the close-up image of porous crumpled MXene sphere in the green box. **d** Pressure response value curve and sensitivity with pressure, inset shows the linear range of pressure response curve. **e** Real-time resistance curve at different pressures. **f** The performance comparison of resistive pressure sensors based on MXene and graphene. **g** Real-time resistance curve of 41.67 kPa pressure load with 1000 recycles, inset shows the enlarged curve from 2000 to 2050s. **h** Pressure response time at a loading pressure of 55.56 kPa

The hollow structure of porous crumpled MXene spheres provided a large specific surface area, which enabled excellent NO_2_ sensing performance and changed its conductivity under external pressure by readily collapsing the structure. We mixed porous crumpled MXene spheres and MXene films and produced a composite membrane by vacuum filtration, which can obtain the ultra-high pressure-sensing properties that pure MXene membrane does not possess. As shown in Fig. 4a, the transient pressure sensor can be fabricated quickly by encapsulating a porous crumpled MXene spheres (MS-2-10) composite membrane between the MXene square electrodes. Figure 4b–c displays the cross section morphology of the composite membrane. It shows many porous crumpled MXene spheres among MXene films, producing a larger interspace for pressure regulation than pure MXene membranes (Fig. S8).

The response values of the sensor under different intensities of pressure are showed in Fig. 4d. The rate of increase in the response gradually slowed down as the intensity of pressure increased. The sensors maintained the highest linear sensitivity of 3.14 kPa^−1^ in the range of 0.14 to 22.22 kPa and only 0.22 kPa^−1^ in the range of 22.22–140 kPa. In a small pressure range, the external force applied to the sensor deformed porous MXene crumpled spheres, increasing the conductive path above and below porous MXene crumpled spheres and lowing the resistance of pressure sensors. When the pressure reached a large pressure range, however, porous MXene crumpled spheres cannot be further compressed as the pressure rises. The applied pressure can only further reduce the interspace between MXene films and porous MXene crumpled spheres, but the compression was more difficult and the variation was tiny. So the sensitivity of the pressure sensor was divided into two parts as the pressure increased. Figure 4e shows the real-time change of the resistance at different pressure intensities. When the same intensity of pressure was loaded three times in a row, the response was almost the same and enhanced with increasing the intensity of pressure. Specifically, a saturated response value of 91.56 was achieved at 555.56 kPa, while a response of 2.22 was obtained at a very low pressure of 140 Pa, indicating an outstanding ultra-wide range of pressure tests. Thus, the sensitivity of pressure sensors based on porous crumpled MXene spheres was not outstanding because the pressure load would reduce the sensor's resistance, and the response range can only be measured between 0 and 100. However, pressure sensor based on porous crumpled MXene spheres shows the widest linear detection range of 0.14–22.22 kPa (Fig. 4f) compared to the previously reported pressure sensors based on MXene and graphene [14, 38–45]. We tested the effect of load residence times on the sensor of 0.1, 0.5, 1.0, 2.0, and 5.0 s. As shown in Fig. S9, the width of the response peak of the real-time resistance curve increased as the loading time lengthened.

Meanwhile, we measured the effect of loading frequency on the performance of sensors under 22.22 kPa pressure load (Fig. S10). With the increase in loading frequency, the response value remained stable to be about 52.5, which proved that the sensor could work stably under different pressure loading frequencies. Immediately afterward, we carried out an anti-fatigue test on the sensor. By continuously applying a load of 41.67 kPa 1000 times, we observed the response was consistently stable around 59 (Fig. 4g), confirming a stable and robust operation even in multiple consecutive tests. The accurate response to the loading times again demonstrated the high sensitivity of the sensors to pressure loads. The response time (defined as the time required during 10–90% of the stable resistance change between pressure releasing and loading states) was only 34 ms at a load of 55.56 kPa (Fig. 4h), ensuring a real-time sensing response to the pressure load.

**Fig. 5:**
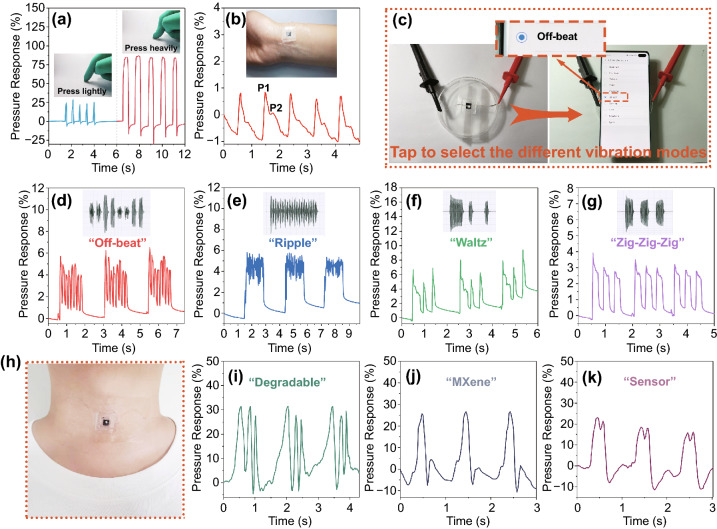
**a** The response results of manual press on the sensor. **b** Real-time recording of the radial artery blood pressure waveform, inset shows the photograph of the wearable sensor attached to the human wrist. **c** Photos of the vibration test of the mobile phone (Samsung S10 Plus). Real-time resistance changes of the pressure sensor during detecting the four different vibration modes of the mobile phone: **d** “Off-beat,” **e** “Ripple” and **f** “Waltz” and **g** “Zig-Zig-Zig.” **h** The photo of the pressure sensor in the throat. The response signal waveform of the pressure sensor toward sounds: **i** “degradable,” **j** “MXene,” and **k** “Sensor”

The pressure sensor in the actual application scenarios was examined when the experimenter presses manually in the low-pressure range in Fig. 5a. The sensor was attached to the wrist of the 25-year-old experimenter through the medical polyurethane (PU) membrane (inset of Fig. 5b) to record radial artery blood pressure. Figure 5b displayed the regular and repetitive waveforms of the wrist pulse with a periodic beating of 96 beats per minute, where the characteristic systolic peak (*P*1) and diastolic peak (*P*2) were observed [46, 47]. Another proven application was the detection of tiny vibrations by sensors. In Fig. 5c, we used the pressure sensors to detect the different vibration patterns of the mobile phone (“Off-beat,” “Ripple,” “Waltz” and “Zig-Zig-Zig” vibration patterns in Samsung S10 plus). The phone was placed flat on the sensor with the sensor in the center of the phone. Then, the experimenter pressed different vibration modes, which were automatically repeated three times (Video S1). It can be observed that the signal waveform of the sensor output was highly consistent with the vibration sound waveform in Fig. 5d–g, which confirmed the capability to respond to tiny vibrations. Based on the performance of sensors, we aggressively tried to simulate the sensors as an electronic throat to receive the sound signal by detecting the motion of the throat and the vibration of the vocal cords [48]. Similarly, the sensor was tightly attached to the throat of the experimenter (Fig. 5h), and then, the experimenter spoke three words (“degradable,” “MXene” and “sensor”) as smoothly as possible and repeated each word three times. In Fig. 5i–k, as a triphthong word, the response signal waveform of the word “degradable” had three peaks, while as diphthong words, the response signal waveform of words “MXene” and “sensor” had only two peaks. In addition, because the pronunciation of the word “MXene” brought greater throat peristalsis than the word “sensor,” the response signal waveform of the word “MXene” was more undulating than that of the word “sensor.” Therefore, the sensors can predict the speech content by recording throat peristalsis and vocal cord vibration to a certain extent. After further improvement and system integration, it is expected to help some mute people.

**Fig. 6:**
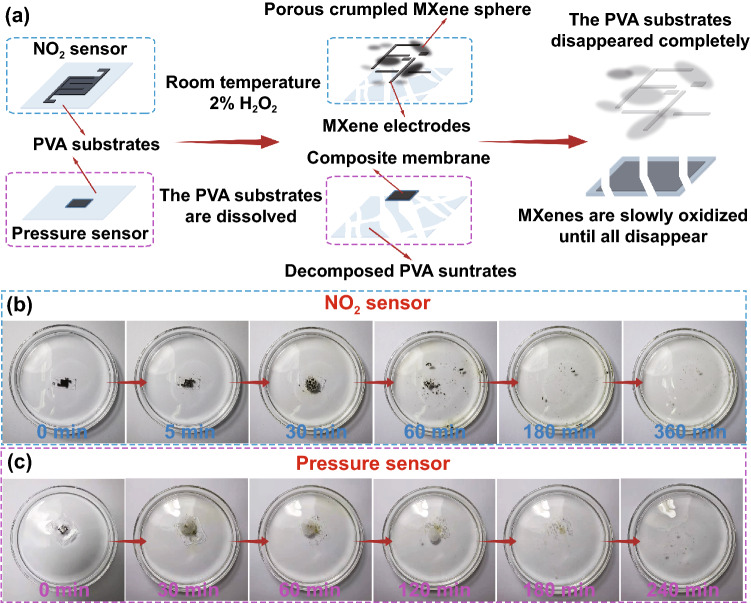
**a** Schematic diagram of the degradation process of the transient NO_2_ and pressure sensors based on MS-2-10. **b** The degradation process of the transient NO_2_ sensor in 2% medical H_2_O_2_ for 6 h. **c** The degradation process of the transient pressure sensor in 2% medical H_2_O_2_ for 4 h

In order to study the degradable performance of the transient all-MXene gas and pressure sensors based on porous crumpled MXene spheres, the sensors were placed in watch glasses with 50 mL H_2_O_2_ of different concentrations, and the state of the sensors was continuously observed and recorded. Figure 6a displays the degradation process of the gas and pressure sensor in 2% medical-grade H_2_O_2_. The PVA substrate was rapidly dissolved within 60 min, while porous crumpled MXene spheres and MXene electrodes were also slowly disappeared after 6 h degradation with the help of H_2_O_2_ in Fig. 6b. The pressure sensors showed a faster full-degradation (4 h) than the gas sensor in 2% medical-grade of H_2_O_2_ (Fig. 6c). The sensors could be rapidly degraded in 10% and 30% H_2_O_2_ for only 30 and 60 min, respectively (Fig. S11), indicating their capability toward controllable degradation. These results fully proved that the transient all-MXene gas and pressure sensors were degradable without any environmental footprint.

## Conclusions

In summary, we have demonstrated the transient all-MXene sensors based on porous crumpled MXene spheres, which took advantage of the unique properties of MXene and the novel structure to achieve excellent gas- and pressure-sensitive performance, respectively. The fabricated NO_2_ sensor exhibited high selectivity, low detection limit (50 ppb), and high response (12.11% to 5 ppm NO_2_). The assembled pressure sensor showed an ultra-wide linear detection range of 0.14–22.22 kPa with a sensitivity of 3.14 kPa^−1^, fast response (34 ms), and excellent repeatability (over 1000 cycles). The pressure sensor attached to the human body can effectively detect the physiological data signals from the wrist pulse signal and the throat vibration during speaking. The multi-functional wearable sensors show excellent controllable transiency with a degradation profile within 6 h in medical H_2_O_2_ (2%). These results highlight the feasibility of realizing wearable multi-functional all-MXene sensors, thus promoting the application of MXene under multiple scenarios and providing new ways to manufacture high-performance, wearable, and degradable sensors required for the Body Internet of Things.

## Supplementary Information

Below is the link to the electronic supplementary material.

Supplementary file1 (MOV 16629 KB)

Supplementary file2 (PDF 1020 KB)
