# Supplementary material for: Self-Assembly 3D Porous Crumpled MXene Spheres as Efficient Gas and Pressure Sensing Material for Transient All-MXene Sensors
Source: Nanomicro Lett. 2022 Feb 5;14:56. doi: 10.1007/s40820-022-00796-7 (PMC8816976; doi:10.1007/s40820-022-00796-7)
Supplement: Supplementary file 2 — Supplementary file2 (PDF 1020 KB) [file 40820_2022_796_MOESM2_ESM.pdf]

Supporting Information for

Self-Assembly 3D Porous Crumpled MXene Spheres as Efficient Gas and Pressure Sensing Material for Transient All-MXene Sensors

Zijie Yang[1], Siyuan Lv[1], Yueying Zhang[1], Jing Wang[2], Li Jiang[1], Xiaoteng Jia[1, *], Chenguang Wang[1], Xu Yan[1], Peng Sun[1], Yu Duan[1], Fangmeng Liu[1, *], Geyu Lu[1]

[1]State Key Laboratory of Integrated Optoelectronics, College of Electronic Science and Engineering, Jilin University, 2699 Qianjin Street, Changchun 130012, People's Republic of China

[2]School of Electronic and Information Engineering, Changchun University of Science and Technology, Changchun 130022, People's Republic of China

*Corresponding authors. E-mail: liufangmeng@jlu.edu.cn (Fangmeng Liu), xtjia@jlu.edu.cn (Xiaoteng Jia)

Supplementary Figures

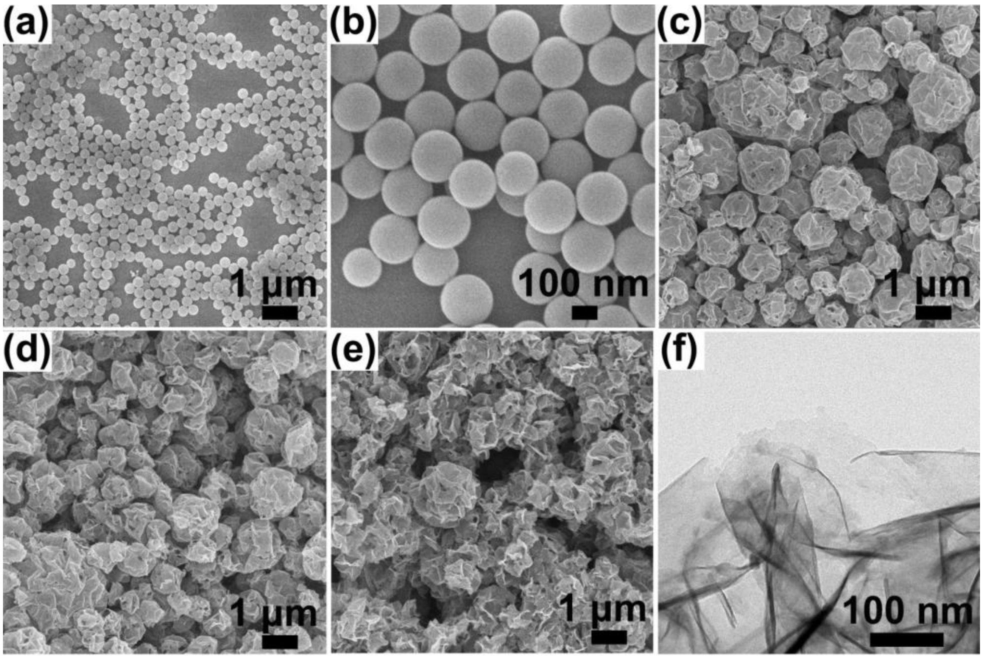

Fig. S1 SEM images of **a-b** PS spheres, **c** MS-2-5, **d** MS-2-10, and **e** MS-2-20. **f** TEM image of MS-2-10

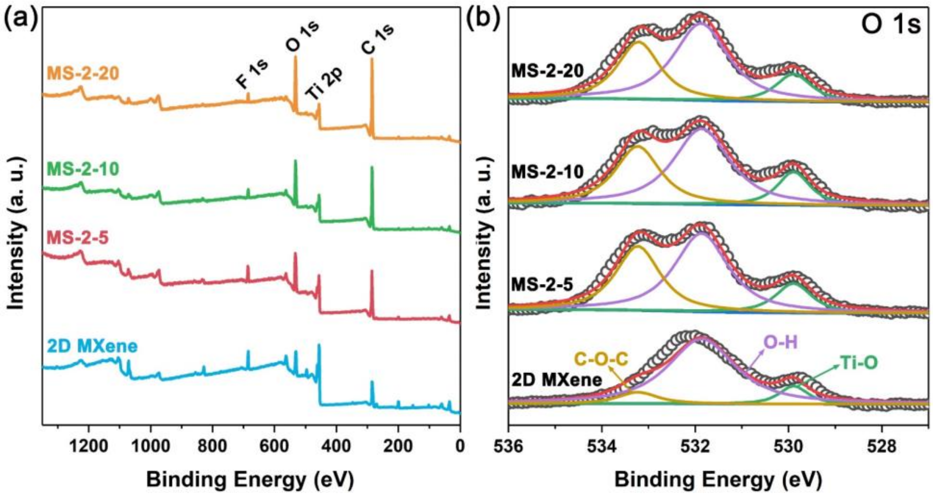

Fig. S2 a XPS survey spectra and **b** O 1s spectra of 2D MXene, MS-2-5, MS-2-10, and MS-2-20

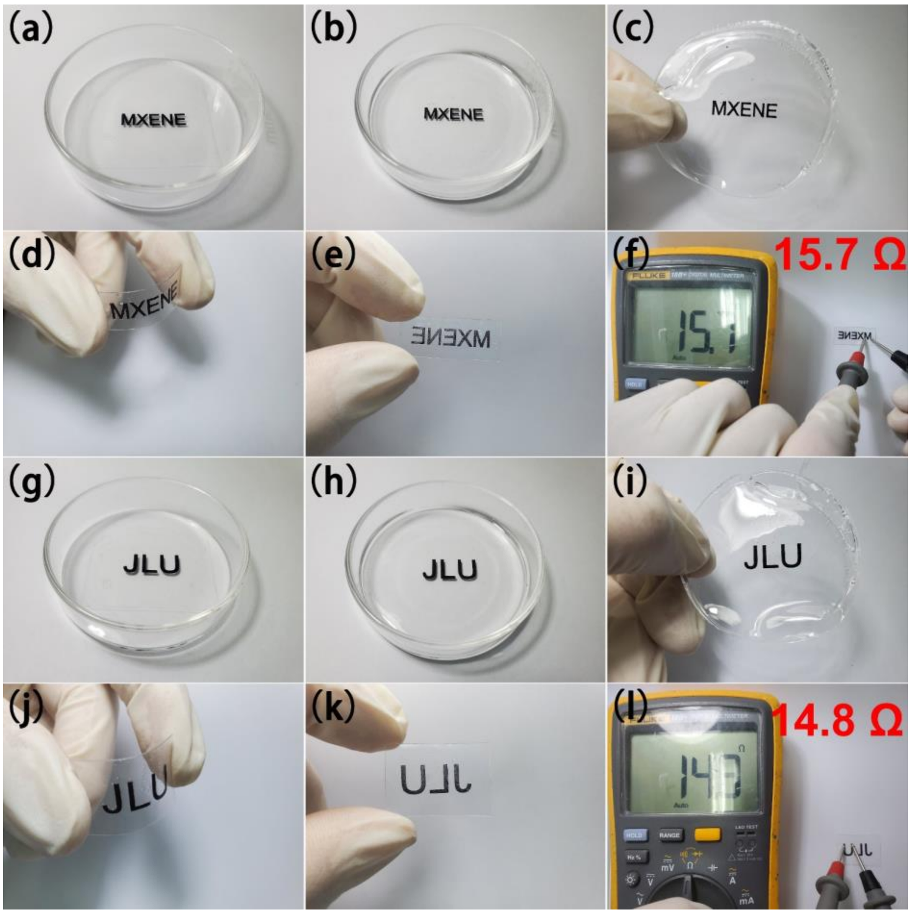

Fig. S3 a-e The production process of PVA substrate with "MXENE" pattern. **f** The resistance measurement at both ends of "M". **g-k** The production process of PVA substrate with "JLU" pattern. **l** The resistance measurement at both ends of "U"

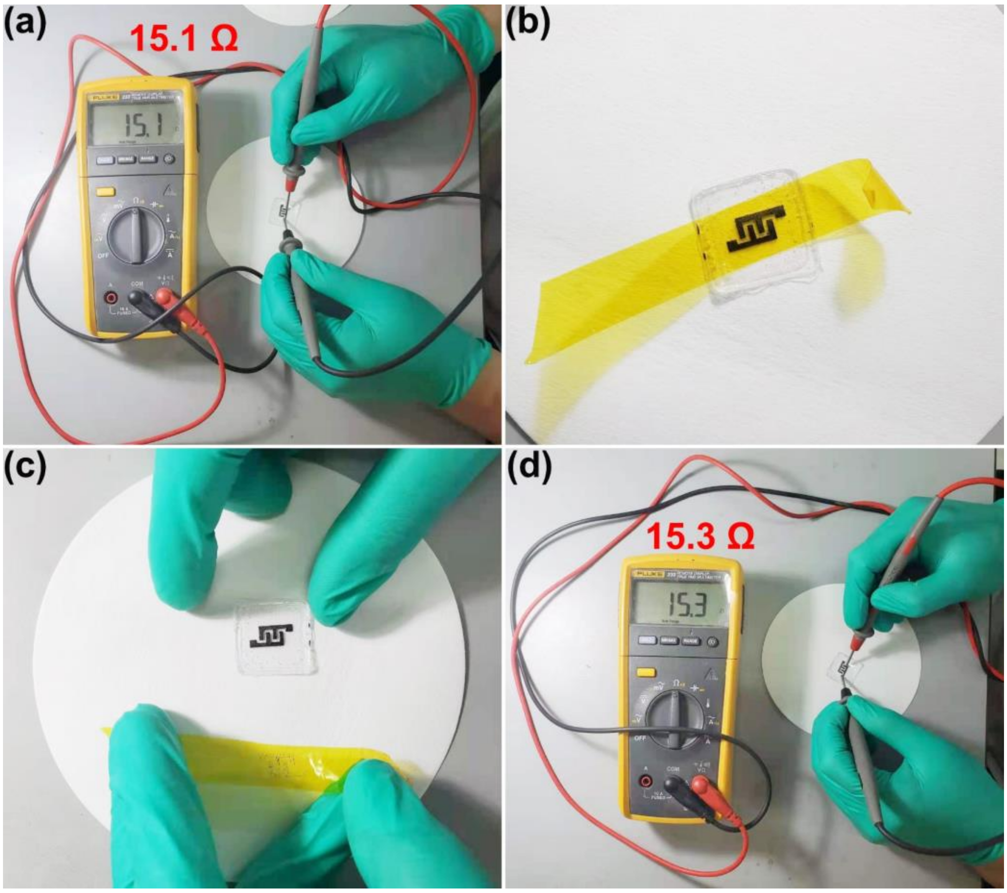

Fig. S4 a The resistance measurement of MXene electrode before sticking tape. **b** The image of MXene electrode with tape. **c** The image of MXene electrode after removing the tape. **d** The resistance measurement of MXene electrode after removing the tape

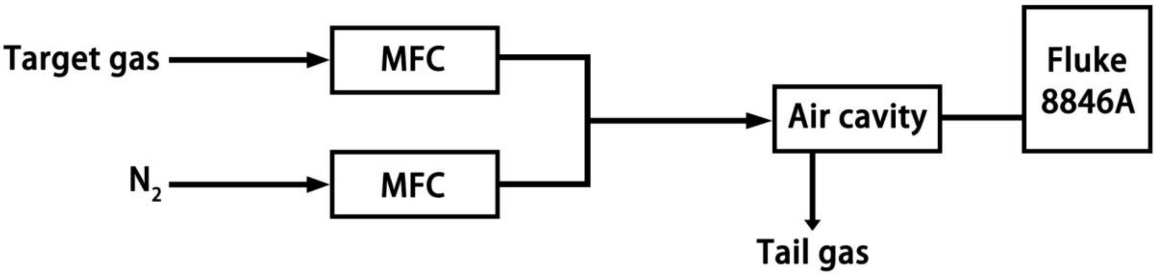

Fig. S5 Schematic diagram of the dynamic test system of gas sensing performance

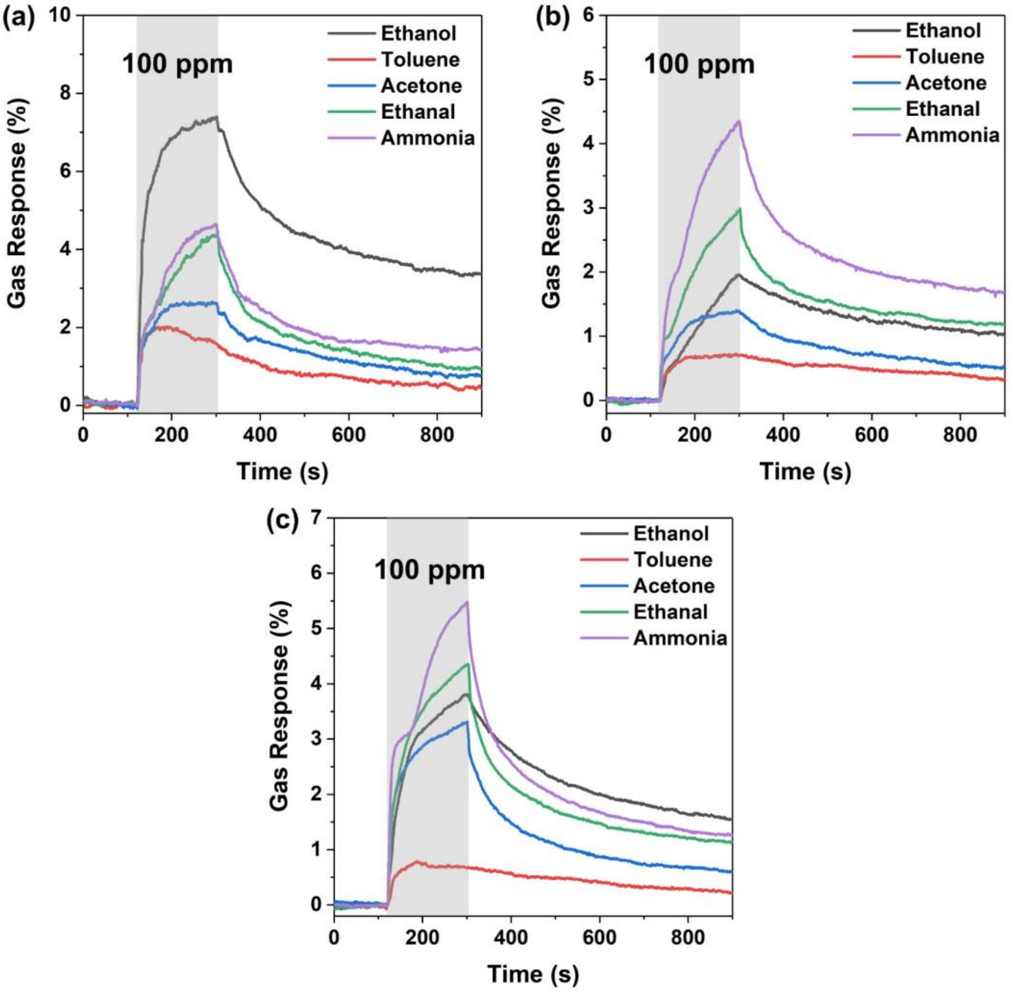

Fig. S6 TDynamic response-recovery curve of sensors based on **a** MS-2-5, **b** MS-2-10, and **c** MS-2-20 upon exposure to 100 ppm of ethanol, acetone, ethanol, toluene and ammonia

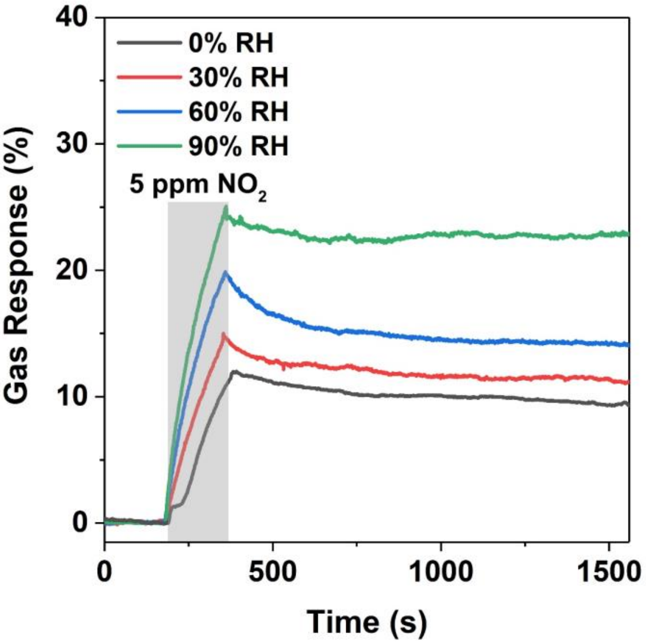

Fig. S7 Dynamic response-recovery curve of sensors based on MS-2-10 upon exposure to 5 ppm NO_2 at relative humidity of 0%, 30%, 60%, and 90%

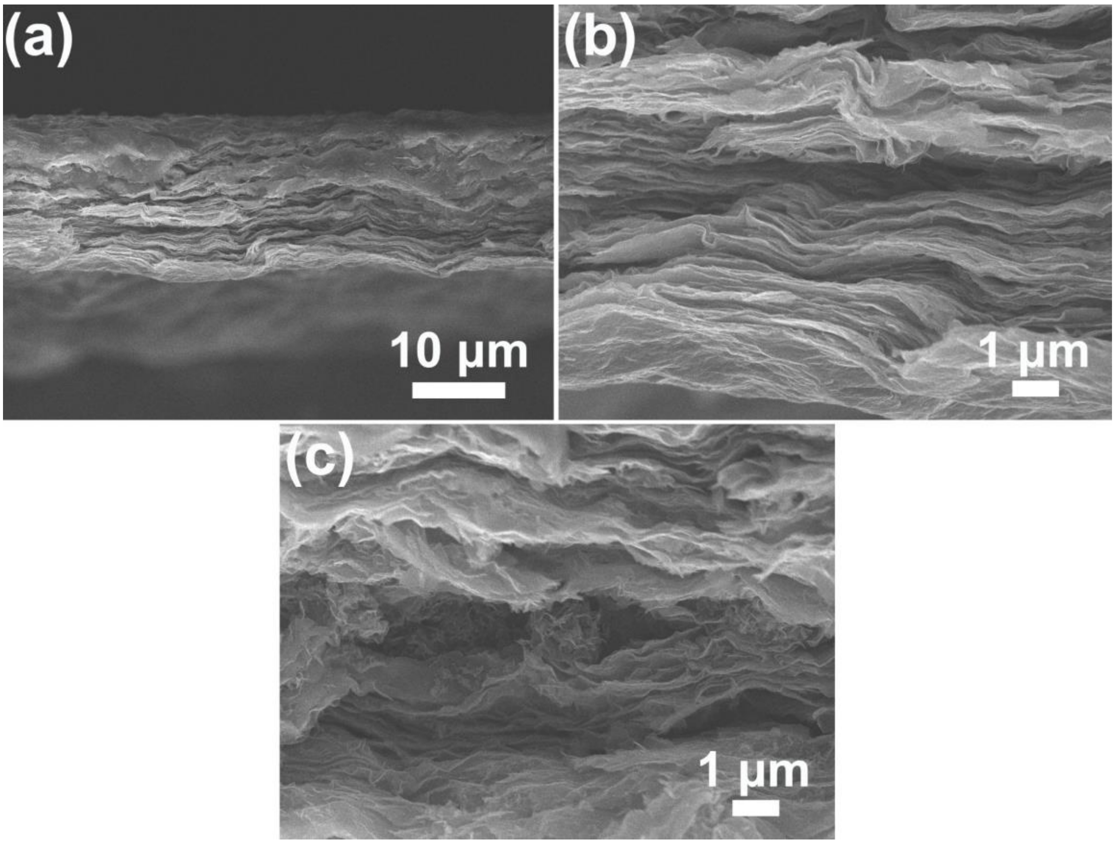

Fig. S8 The cross-section SEM images of **a-b** pure MXene membrane and **c** the composite membrane

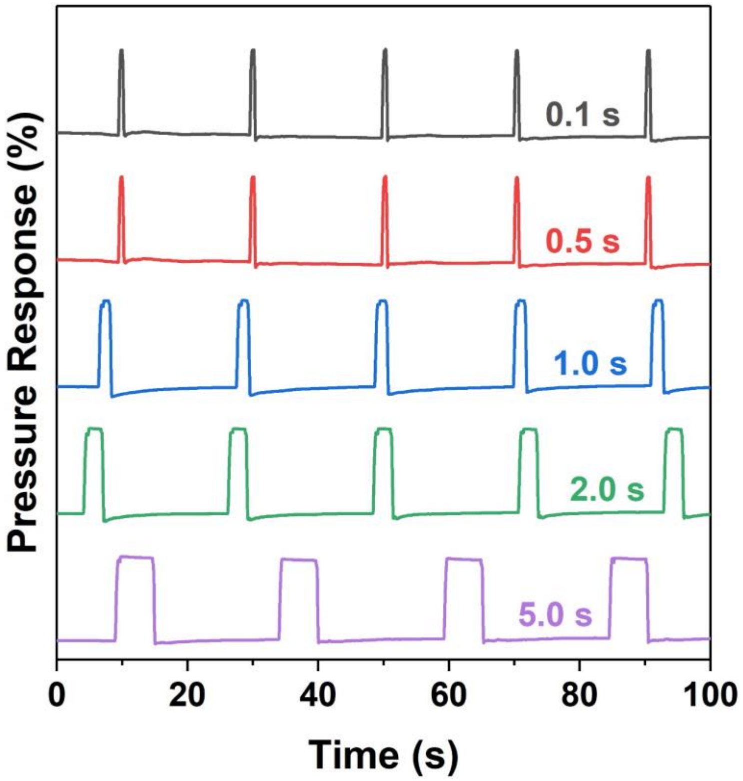

Fig. S9 Real-time resistance curve of pressure sensor on 22.22 kPa load with different duration

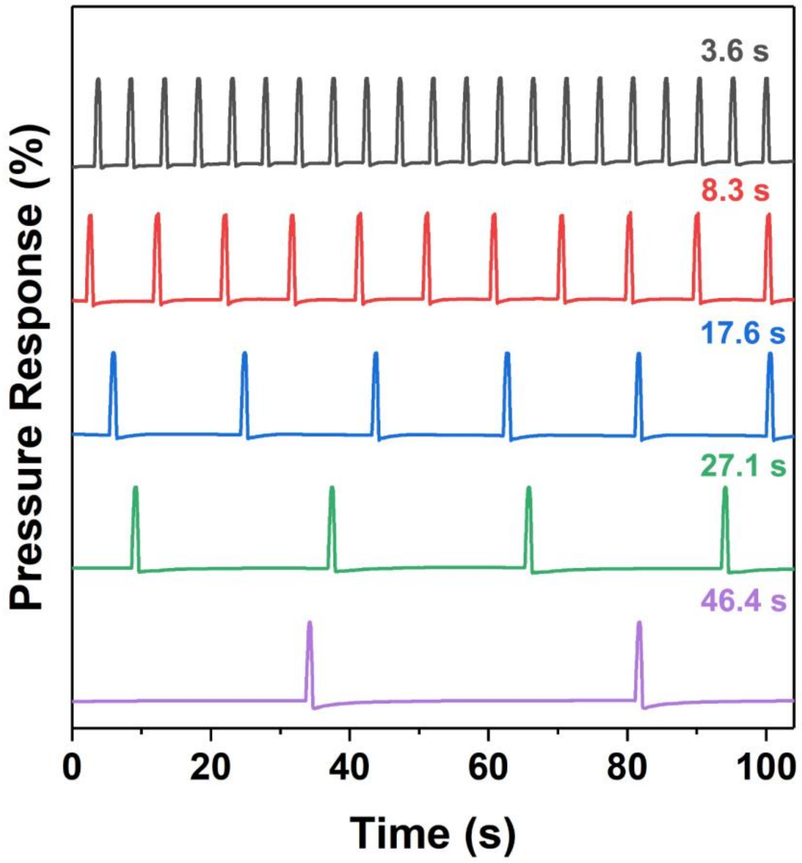

Fig. S10 Real-time resistance curve of pressure sensor on 22.22 kPa load at different intervals

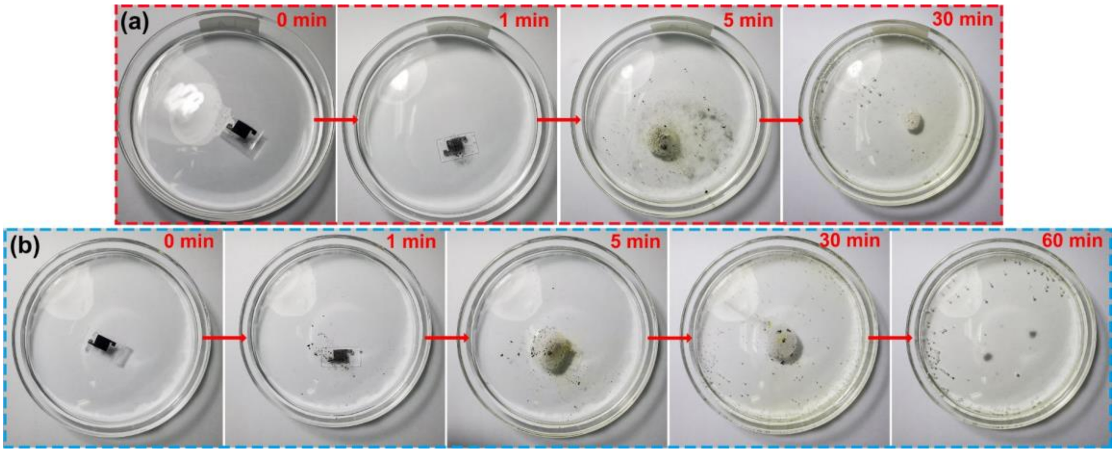

Fig. S11 a The degradation process of the transient NO_2 sensor in 30% H_2O_2 for 30 min. **b** The degradation process of the transient pressure sensor in 10% H_2O_2 for 1 h
